# A review of the value of innovation in inhalers for COPD and asthma

**DOI:** 10.3402/jmahp.v3.28760

**Published:** 2015-09-16

**Authors:** Johann Christian Virchow, Cezmi A. Akdis, Josep Darba, Richard Dekhuijzen, Sylvia Hartl, Gisela Kobelt, Albert Roger, Steven Simoens, Mondher Toumi, Ben Woodhouse, Adam Plich, Saku Torvinen

**Affiliations:** 1 Department of Pneumology/Intensive Care Medicine, University of Rostock, Rostock, Germany; 2 Christine Kühne-Center for Allergy Research and Education (CK-CARE), Swiss Institute of Allergy and Asthma Research (SIAF), University of Zurich, Davos, Switzerland; 3 Department of Teoria Econòmica, University of Barcelona, Barcelona, Spain; 4 Department of Pulmonary Diseases, Radboud University Medical Centre, Nijmegen, The Netherlands; 5 Department of Respiratory and Critical Care, Otto Wagner Hospital, Vienna, Austria; 6 European Health Economics, Mulhouse, France; 7 Allergy Unit, University Hospital Germans Trias I Pujol, Badalona, Spain; 8 Department of Pharmaceutical and Pharmacological Sciences, KU Leuven, Leuven, Belgium; 9 Department of Public Health, University of Marseilles, Marseille, France; 10 NHS Bolton Clinical Commissioning Group, Bolton, UK; 11 TEVA Pharmaceuticals Europe B.V., Amsterdam, The Netherlands

**Keywords:** Asthma, Chronic obstructive pulmonary disease, innovation, patient preference, adherence, patient compliance, metered dose inhaler, dry powder inhaler, medication errors, value

## Abstract

**Background:**

Appropriate use of inhaled therapies for asthma and chronic obstructive pulmonary disease (COPD) is critical to ensuring good patient outcomes, efficient use of healthcare resources and limiting the effects of high-morbidity. The appropriate choice of inhaler and active therapy, incorporating patient preferences, can help improve treatment adherence and long-term outcomes. Despite this, many current inhalers are non-intuitive to use, and require extensive training.

**Methods:**

In this review, an expert panel considers the evidence for the use of inhaler devices in management of COPD and asthma. The panel also evaluates the value of innovation in inhaler technologies, which optimise the use of existing molecules from a clinical, economic and societal perspective.

**Conclusions:**

The panel conclusion is that there remains a substantial unmet need in inhaler technology and that innovation in inhaler devices can provide real-world health benefits to patients. Furthermore, we recommend that these innovations should be supported by healthcare systems through appropriate pricing and reimbursement mechanisms.

Chronic respiratory diseases, such as asthma and chronic obstructive pulmonary disease (COPD), are responsible for more than 150,000 deaths in the European Union (EU) every year and affect over 60 million more (1). This clinical impact is reflected in the economic burden of these diseases in the EU, with the combined annual cost of care and productivity losses amounting to EUR33.9 billion and EUR48.4 billion for asthma and COPD, respectively (2013) (1). Against this backdrop of high morbidity, mortality, and economic burden, the goals of treatment in asthma and COPD are to optimise disease control, with limited flare-ups and rare occurrence of severe exacerbations (2, 3). Although oral, injectable, and inhaled products are available, inhaled therapy is preferred because of the high drug concentration which can be achieved locally within the lungs, with reduced risk of systemic side effects (4). However, good-quality outcomes with inhaled therapy hinge upon the appropriate use by patients (4). A range of inhalers are available to deliver therapy, each of which is associated with benefits and drawbacks. In general, inhalers can be divided into two categories: pressurised metered dose inhalers (pMDI) and dry powder inhalers (DPI). Within and between these categories the inhalers differ in effectiveness of drug delivery and ease of use (5).

Global clinical guidelines issued by the Global Initiative for Asthma (GINA) and the Global Initiative for Chronic Obstructive Lung Disease (GOLD) recommend that persistent, moderate-to-severe asthma and COPD at high risk of exacerbations be treated with a combination therapy of inhaled corticosteroid (ICS) and long-acting beta-agonist (LABA), administered via an inhaler. The guidelines also recognise that this therapy is best administered in a fixed-dose combination (FDC) from a single inhaler, rather than from two separate inhalers (2, 3). This is demonstrated by reduced adherence and increased discontinuation rates in patients using multiple inhalers versus those using FDC devices (6).

Choosing the appropriate molecule combination and the inhaler are important determinants of treatment outcomes. This choice relies upon matching the needs of a particular patient to the benefits of a particular inhalation device. In addition to considering a patient's disease stage, the choice of treatment should also be influenced by the patient's ability to administer the prescribed dose effectively and the device preferences. This is important, because misuse of inhalers will have a significant negative impact on disease control (7, 8). As the dose delivered to the lungs is highly dependent on the correct use of the delivery system, the European Respiratory Society recommends that those who prescribe inhalers should ensure that patients use their therapy correctly (5). This is particularly important as training patients on the correct use of many existing inhaler devices can be difficult, leading to errors and low patient satisfaction.

The availability of novel inhalers has been facilitated by the loss of exclusivity rights for several of the molecules that form the basis of inhaled therapy for asthma and COPD. However, national healthcare systems in several countries (e.g., Germany, Austria, Belgium, Poland, Romania, Slovakia, Hungary, and Lithuania) do not recognise the value of innovation in new inhalers through their pricing and reimbursement decisions, which may result in restricted availability of innovative inhalers in these countries. The lack of access limits the options to optimise patient care, and may discourage further investment in inhaler development.

In this paper, we examine some of the key benefits of inhaler innovation that support the rationale for investment in improved inhaler devices. This expert panel review of the available evidence postulates that the value of clinically meaningful innovation in inhalers, with off-patent molecules, should be recognised through pricing and reimbursement decisions. This innovation must deliver real-world health benefits to patients, which must address clinically relevant unmet needs.

## Methodology

A wide range of literature has been published on the effect and impact of innovations in asthma and COPD and establishes the role of appropriate, well-used inhaled therapies. In order to fully understand the complex features of the treatment of asthma and COPD, a two-stage process was adopted.

### Semi-structured literature review

In the initial stage, a semi-structured literature review was conducted to understand the scope and issues associated with various aspects of asthma and COPD therapy. Broad search terms were applied to establish a full review of the (English language) literature using PubMed. The terms used were asthma, COPD, adherence, compliance, persistence, patient, preference, choice, economics, social, and outcome. A rapid review was conducted to remove all references not related directly to inhaled therapies for asthma and/or COPD, duplicate references/corrections, publications on highly specific/atypical patient populations, and commentary publications on other studies. A final shortlist of 428 papers was used to create materials taken forward to the Delphi panel review. These materials summarised the 428 papers into seven critical dimensions as follows: current unmet needs, patient compliance and adherence, patient preference, choice and outcomes, safety and pharmacovigilance, access to innovation, and economic impact of innovation. Within each dimension, four or five proof points were developed to present key supporting evidence.

### Delphi panel review

A face-to-face panel was convened consisting of eight experts from diverse backgrounds (clinical, economic, and social) and a number of different EU countries. The goals of the panel were to objectively assess the existing evidence regarding inhaler innovation. All experts that participated in the face-to-face panel are the authors of this review.

Over 2 days, the panel was presented with the summary statements for each of the seven critical dimensions identified during the semi-structured literature review. The panel discussed each summary statement and supporting proof points in detail. Subsequently, an anonymous Delphi evaluation (9) was conducted using the following approach: for each summary statement, the panel was presented with a short overview of the evidence base and asked to comment anonymously on the summary statements and proof points (in terms of language, importance, and strength of supporting data) and to recommend, again anonymously, enhancements to the data set and the summary of the data.

For each summary statement, the anonymous feedback was aggregated and fed back; subsequently, one to four rounds of anonymous feedback and iteration were completed to establish a consensus on the best summary of the evidence provided. This consensus has provided the expert perspectives used throughout this review. Sections within the perspectives section reflect the consensus summary statements agreed by the panel.

## Perspectives of the expert panel

### Current inhalers are often poorly used, are not intuitive, and require extensive training

Current pMDI and DPI inhalers require a complex administration procedure involving several steps (dose loading, inhaler priming, and breathing management) in order to ensure maximal benefit of the medication. These steps require that patients display great dexterity and coordination (10). Failure to follow the instructions may lead to inhalation errors, some of which reduce, or prevent entirely, deposition of the medication in the lungs (11). In a videotaped study using a validated scoring technique, 40% of COPD patients demonstrated at least one error in technique (12).

In addition to problems in achieving correct inhalation technique among the general patient population, several specific groups have particular problems using existing inhalers. This leads to certain patients, such as those with low inspiratory flow rate, poor cognitive ability (13), arthritis, and the elderly, having difficulty achieving adequate dosing (14, 15). Incorrect inhalation technique is particularly common among older people and those with reduced inspiratory flow rate (15). Indeed, most patients with COPD are unable to use a pMDI or DPI correctly. Common errors include inadequate coordination of inspiration and actuation, and inability to achieve a sufficient inspiratory flow rate (15).

Complicating this situation is the fact that the capacity for physicians and nurses to train the patient is often limited. Both time constraints and inadequate knowledge among healthcare professionals (HCP) can lead to ineffective patient education. In a review of 20 relevant studies, only 28% of doctors and 22% of nurses were able to describe or perform all the critical steps for using inhalers (16). Many patients do not receive inhaler training of any kind, with one study showing 25% of patients report having never received verbal instruction on correct inhalation technique (17).

Poor training, coupled with complex procedures for use, mean that 50–90% of asthma patients show incorrect technique in clinical studies (18).

Even when patients are able to demonstrate correct inhaler technique during consultation with an HCP, they may not maintain this standard at other times (19, 20). Although retraining on inhaler technique can help some patients, 65–78% of patients who show poor technique on pMDIs do not improve with subsequent training (21).

### Difficulties of using current inhalers can negatively impact patient outcomes through patients displaying poor inhalation technique

The frequently cumbersome nature of existing inhalers makes patient education and disease management more complex and reduces healthcare efficiency (4). Correct use of inhalers is critical to ensuring that patients receive their prescribed medication doses. In a recent survey, 66% of expert physicians cite ‘failure to master device’ as a primary reason for lack of efficacy in respiratory disease (22). This belief is supported by real-world observations of both reduced lung deposition of the active ingredient and worsening of asthma control as the number of mistakes in inhaler technique increases (23). Failure to use inhalers correctly can significantly impact disease management (2, 24).

Poor inhalation technique, as measured by the occurrence of critical inhaler errors, has been shown to significantly reduce the degree to which asthma and COPD are controlled, to impact patient health-related quality of life (HRQoL), and to increase the risk of unscheduled healthcare resources being required for disease management. Melani and colleagues found that patients making critical inhaler errors were at significantly higher risk of suffering limitations in everyday life, shortness of breath, use of reliever inhalers, uncontrolled disease, and sleep disturbance (*p*<0.009). Furthermore, the occurrence of critical inhaler errors also led to significant increases in the risk of requiring hospitalisation, visiting the emergency departments, requiring antibiotics, and using a course of oral corticosteroids (25).

In a second study, asthma stability, as measured by the asthma instability index score (AIS), has been found to be impacted by poor inhaler use in France. AIS and evaluation of inhalation technique data were available in 3,709 asthma patients (out of a total evaluable population of 3,955) and showed that people who did not use their inhalers correctly recorded significantly higher AIS scores (7).

Incorrect use of inhalers, including under- and over-dosing driven by patient error, can also impact safety and tolerability (25). Inappropriate patient use has also been cited as a primary contributing factor in adverse events (25).

### Choice of inhaler, taking patient preference into account, can support optimal disease control

In addition to the impact on critical errors, inhaler complexity, confidence in efficacy, and the need for frequent retraining can influence patient preference for specific inhalers. Consequently, many patients express preferences for certain devices, and those who are able to use their inhaler efficiently are more likely to express a preference for the inhaler they currently use (26–29). Patients’ perceived satisfaction with their inhalers in asthma correlates with improved disease control and treatment adherence (29). Patients have expressed a preference for an inhaler that is small, with an ergonomic mouthpiece and an easy to use dose preparation mechanism, and providing enough medicine for at least a month of treatment (30).

It has been shown that increases in adherence, quality of life, and disease control (including exacerbations and hospitalisations) are directly linked to patients’ satisfaction with their inhaler device (28–31). Conversely, poor patient preference, coupled with the complexity of inhaler use and the implied critical errors, adversely impacts adherence to the prescribed regimen (29).

In addition to real-world evidence that effectiveness can be maximised by using a patient-preferred inhaler, a critical element at the heart of choice of therapy is the maintenance of patient safety. This is particularly important for inhaled medicines, where the complexities of getting the right amount of drug to the lungs can impact the risk–benefit profile of the drug–device combination (32).

Finally, patient preference can also drive healthcare resource benefits. Several cross-sectional studies have shown that, even if the inhaler is more expensive than standard inhalers, selecting an inhaler based on the patient's preference can be cost-effective (27). In one study in which 100 patients were randomised across seven different devices, it was demonstrated that using a patient-preferred inhaler could save 14% of total costs associated with asthma (27).

A greater range of treatment options in inhalers brings opportunities for tailoring healthcare to individual patient needs, through physician/patient dialogue. Offering patients and providers choice also helps to create competition, encouraging efficiency and responsiveness to patients’ preferences and healthcare needs.

### Poor adherence to asthma and COPD treatments is associated with an increased number of exacerbations, hospital admissions, and deaths

For inhaled therapy in chronic lung disease, non-optimal usage can take two forms; firstly, patients can fail to initiate treatment which can be related to the ease of use of a particular inhaler. Secondly, the patient can fail to follow their treatment regimen as prescribed, for example, by missing scheduled doses or by stopping treatment after a period of time. Even after accounting for the context of chronic diseases, where adherence rates are typically between 60 and 80%, poor adherence has been observed in respiratory disease (33). On average, adherence rates for asthma and COPD are approximately 50–60% (34).

Real-world evidence demonstrates that poor adherence to asthma and COPD treatment is associated with an increased number of exacerbations, hospital admissions and deaths. Approximately one in four asthma exacerbations are attributable to poor adherence (35). In addition, for asthma patients on FDCs, each 25% increase in adherence rate reduces an asthma related hospital visit by 10% and the odds of needing additional reliever therapy (short-acting beta agonist) by 10% (36). In a study of more than 30,000 adults, the rate of mortality linked to asthma decreased by 21% for each additional inhaler prescribed in the prior 12 months (demonstrating that better compliance can reduce mortality) (37). A similar study of over 6,000 patients demonstrated that adherence to inhaled medication is significantly associated with reduced risk of death and admission to hospital due to exacerbations in COPD (38).

Inhaler ease of use and patient satisfaction can improve adherence and enhance long-term efficiency in the use of healthcare resources (35, 36). In a real-world observational study of asthma specialists (*n*=330), primary care physicians (*n*=252) and asthma patients (*n*=2,135), increasing patient satisfaction with inhaler correlated with improved treatment adherence (29). Similar findings were reported for 1,443 real-world COPD patients (39). These data suggest that providing patients with a simple, easy-to-use, effective inhaler, has the potential to increase patient treatment adherence and therefore to improve patient outcomes.

Improving treatment adherence can also reduce healthcare costs associated with the treatment of asthma and COPD. A retrospective analysis of a claims database of 1,365 COPD patients in Spain showed that non-adherent patients incurred higher medicine costs than adherent patients (40).

### Different inhalers have different dose-delivery profiles and cannot be directly substituted

It is important to ensure that patients always receive an appropriate dose of medication when they use their inhaler. Inconsistent dosing occurs for a number of reasons that can be either inhaler or user dependent. The amount of drug delivered to the lungs depends on the patient's ability to use their inhaler correctly and to avoid making inhaler errors; it also depends on the technical characteristics of the inhaler and drug formulation (41).

Many inconsistencies in dosing occur as a result of patient errors. In the case of pMDIs, poor coordination and the patient's breathing rhythm have been identified as the main reasons for inconsistent dosing (19). Along with the coupling of inhaler and treatment, other features of an inhaler may also lead to inconsistent dosing. Many inhalers require the patient to clean any excess powder from the inhaler mouthpiece (42). Otherwise dosing can become inconsistent, due to dose accumulation taking place in the mouthpiece of the inhaler (42).

Regulatory pathways have been established which address the complexity of inhaler dose delivery mechanisms. Inhaled products with off-patent molecules are registered under a comprehensive and complex hybrid approval (Article 10.3 of Directive 2001/83 EC). In order to show therapeutic equivalence, the clinical (pharmacodynamic) data needs to be submitted alongside the typical pharmacokinetic data to support the application as required by the Orally Inhaled Product (OIP) guidelines. The key challenge is showing the therapeutic equivalence given the inconsistency in performance of the original inhalers. The regulatory framework in Europe, using a stepwise approach based on both *in vitro* and *in vivo* data, recognises the complexity of demonstrating equivalence (43).

Even when inhalers successfully meet the therapeutic equivalence criteria set out by the regulatory bodies, account must be taken of real-life practice, where the inhaler can have a strong impact on efficacy for the safety of the individual patient. A patient's ability to use one inhaler does not imply that they will readily and accurately use a substitute. In a 2-year retrospective study in the UK of 824 matched patients, the odds ratio for treatment success was significantly lower in the switched cohort compared with controls (*p*<0.001), while the odds ratio for unsuccessful treatment was significantly higher (*p*<0.001) (44). The authors acknowledge the potential for alternative reasons for this observation but conclude that switches in ICS device without clinical visit or consultation were associated with worsened asthma control (44).

Real-world observational studies show that the level of disease control that can be achieved is at least partly driven by inhaler characteristics and not just by the drug (28). Each inhaler has a specific routine that must be followed and patients need to be adequately trained before a switch may be considered (28). The benefits of choosing an optimal inhaler first time, avoiding wastage and ineffective inhalation, are evident (27). Physicians and their staff must be central to the decision to change inhaler, while providing training and checking technique. A more intuitive inhaler, which can be handled correctly by more patients, may have the potential to reduce costs.

### Innovative inhalers can contribute to improving patient inhaler technique and achieving disease control leading to better allocation of healthcare resources

Investing in innovation in respiratory inhalers delivers economic benefits, both directly within healthcare (through better management of healthcare resources, fewer exacerbations, and improved healthcare efficiencies) and indirectly (through maintenance of productivity, inward investment in skills, and long-term employment).

In 2010, the proportion of treated asthma patients assessed as having ‘not well-controlled’ asthma was 53.5% (45). On average, a patient with uncontrolled asthma costs a healthcare system around four times as much as a patient who is well controlled on therapy (46, 47). A proportion of these costs could be avoided through improved inhaler efficiency and technique.

A recently published economic model in the UK suggested that, in 2013, poor inhalation technique among the users of FDCs of ICS and LABA was substantial. It was estimated that approximately 12% of direct unscheduled healthcare costs of asthma and COPD can be attributed to poor inhalation technique (none planned visits and treatment due to flare up) (48). Rates of acute care episodes are 3.5 times more likely for those with three to four control problems versus those with no control problems (49), while improved adherence is associated with reduced rates of hospitalisation and associated costs (50).

## Discussion

The perspectives outlined above postulated around the benefits that could be achieved with continuous innovation in respiratory devices. There remains an unmet need for new and innovative inhalers to address some of the limitations of existing products. In addition, innovative inhalers should offer attributes and features that patients particularly value, and contain different classes and combinations of medicines used to treat asthma or COPD by inhalation. Difficult-to-use inhalers can negatively impact patient outcomes through patients displaying poor inhalation technique which can contribute to low treatment adherence. Evidence shows that poor inhalation technique is associated with an increased risk of hospitalisations, emergency department visits, and administration of antimicrobials or oral corticosteroids; all of which translates into considerable cost implications while also increasing the risk of side effects and added morbidity. Similarly, low adherence to asthma and COPD treatments contributes to poor patient outcomes and is associated with considerable costs. A lack of satisfaction with existing inhalers is one of the reasons for poor asthma and COPD treatment adherence, as evidence shows that the more satisfied patients are with their inhalers, the more likely they are to adhere to treatment. In that context, patient preference for certain inhalers, or more precisely for certain attributes and features, are important. As the evidence reviewed here shows, patient preference can be a good predictor of future treatment outcomes.

Taking all the above into account, there is potential for new and more effective inhalers to reduce the overall cost burden associated with asthma and COPD. More patients displaying the correct technique and improved patient adherence would ultimately lead to fewer unscheduled healthcare events that lead to increased costs. However, it needs to be questioned whether adherence should be viewed as a patient relevant outcome parameter rather than a variable to explain treatment success. Theoretically, it might be possible that a comparatively easy to use and forgiving inhaler would deliver higher levels of treatment success and therefore prompt patients to decrease its use, which has been called intentional non-adherence. Thus, the interplay between inhaler ease of use, patient preference and satisfaction, adherence, and patient relevant outcomes may be considerably more complex than suggested by simple one-dimensional models of inhaler features determining patient adherence and eventually outcomes.

In the current environment, there are effective medicines but ineffective delivery methods, and these are driven by the complexity and multitude of existing inhalers and inadequate training. Optimising the delivery device represents a primary area of unmet need in ICS/LABA treatments (23, 51). New inhalers need to have simple and intuitive operation. To provide a low rate of accidental critical errors, they must consistently deliver the correct dose across a range of real-life situations, for example, low inspiratory flow rate, varied inhalation-actuation timings, and a range of inhaler orientations (52). This should be preferably delivered in a limited number of steps required to charge and properly use the inhaler such as a handling sequence limited to ‘exhale – open – inhale – close’ manoeuvres. In addition, the inclusion of positive feedback mechanisms is absent in many inhalers. These measures enable patients to feel confident that an appropriate dose has been delivered, while supporting the maintenance of correct inhaler usage (53).

Development of novel inhalers is complex, time consuming, and costly. This is attributable to the strict requirements of the regulatory pathway for hybrid inhaler device medicine combinations; the fact that the production capability for such devices requires significant investment and also the large resources required to commercialise such medicines through investment in physician education and the provision of training. Aside from financial considerations, there are technological barriers to the development of innovative new inhalers, and only a limited number of pharmaceutical companies currently have sufficient knowledge to develop new inhalers. This assertion is supported by the fact that, despite genericisation of the molecules for numerous asthma therapies (e.g., Symbicort in 2011) (54), the costs of development and the uncertainties of access have meant that direct alternatives containing the same active ingredients are limited across the EU.

Patients will only be able to benefit from innovation in inhaler devices if the pharmaceutical manufacturers have sufficient incentives to invest in such innovations. However, mechanisms of cost control may act to restrict the development of improved inhaler technology. In the context of novel inhalers with off-patent molecules, this includes the generic approach to pricing of such medicines by many healthcare systems in Europe as well as attempts to tender them as commodities. Lack of recognition of innovation in inhaler devices which deliver off-patent molecules can, at best, prohibit patients in these countries accessing new inhaler technology. At worst, it can limit the financial incentives for the pharmaceutical manufacturers to invest in the development of novel inhalers.

There are numerous approaches to address the development and introduction of novel inhalers, ranging from the flexibility to combine a full portfolio of medicines to treat asthma or COPD and to choose a conventional pricing and reimbursement process, to the development of a specific process for hybrid medicines with off-patent molecules similar to the approach adopted for biosimilar therapies.

Healthcare systems need to encourage device-led innovation by allowing reimbursement decisions to reflect such innovation. More competition in the field of inhalers will lead to innovation in new inhaler technology and deliver improved health outcomes.

## Conclusion

Innovation in inhaler devices provides real-world health benefits to patients by addressing clinically meaningful unmet needs. As such, with appropriate clinical studies addressing handling and safety issues, it should be recognised through pricing and reimbursement approaches. Access to innovative inhaler devices, at a price that reflects the value of clinically meaningful innovation, has the potential to bring benefits to patients, budget holders, clinicians, and wider society.
